# Evaluation of sperm counting accuracy on computer-assisted sperm analysis with GoldCyto® slides and glass slides

**DOI:** 10.3389/fvets.2023.1283128

**Published:** 2023-10-09

**Authors:** Eser Akal

**Affiliations:** Department of Reproduction and Artificial Insemination, Faculty of Veterinary Medicine, Ondokuz Mayıs University, Samsun, Türkiye

**Keywords:** CASA, glass slide, GoldCyto ® slide, sperm concentration, sperm

## Abstract

Worldwide, various counting chambers and computer-assisted sperm analysis (CASA) devices are in use. The semen’s concentration can vary depending on the depth of the counting chamber and how it is loaded. The study’s objectives were to analyze the effects of various counting chambers on semen concentration results using a GoldCyto® slide and a glass slide in the CASA system and to ascertain the precision of concentration measurements made using glass slides on CASA. The study’s control group was composed of samples with known concentrations (72–80 million sperm/mL) as determined by a spectrophotometer. A total of 21 frozen straws from the same bull of the same date were thawed at 37°C for 30 s and loaded into two different sperm-counting chambers (GoldCyto® slide and glass slide). The sample semen placed in the sperm counting chambers was 5 μL and the same value was entered in the CASA software as 5 μL. Measurements were done and evaluated in 5 different areas. According to the data we obtained, using the glass slide were statistically lower than the spectrophotometer (*p* < 0.001). GoldCyto® slide results were consistent with spectrophotometer results. Consequently, measurements with GoldCyto® slides in the CASA had consistent results, while measurements with glass slides were inconsistent. It was concluded that GoldCyto® slides are more suitable than glass slides in the concentration examinations of semen. Therefore, more study is needed to optimize the use of glass slides.

## Introduction

Semen analysis includes examining the physical properties of semen (color, odor, pH, and viscosity), volume, concentration, morphology, motility, and progression, which is performed repeatedly at different intervals (1). Sperm concentration is one of the most important parameters in determining fertility. Sperm concentration is defined as spermatozoa per unit semen volume and is inversely proportional to the amount of diluent (2). An incorrect assessment of semen concentration affects the breeding station’s output efficiency significantly. The concentration of spermatozoa is critical for optimizing insemination dose and diluting semen. Breeding centers tend to prolong ejaculation as much as possible depending on sperm concentration to increase the number of semen doses production (3). In addition, the total number of spermatozoa per ejaculate and sperm concentration are associated with pregnancy rates and are predictors of conception (4).

Sperm concentration per 1 mL of semen can be determined using a hemocytometer, spectrophotometer, CASA, or flow cytometry (5, 6). Flow cytometry is the most precise method to determine sperm concentration. However, before performing flow cytometry, a preliminary assessment of sperm count by a different method is required to ensure proper semen dilution (close to 250,000 sperm/mL) (7). A popular and definitive method of determining sperm concentration in animal sperm is spectrophotometry. Spectrophotometers have been developed to assess sperm concentration as an alternative to the technically more difficult and time-consuming use of hemocytometers. However, it may not be an appropriate method for evaluating low-concentration and low-volume sperm samples (8, 9).

CASA, a new method for sperm evaluation and concentration measurement, has been developed recently. This method uses a video camera attached to a microscope to analyze images for concentration. CASA allows for fast, low-cost, and highly accurate calculation of sperm concentration, but precision is marred by several technological challenges and deviations (10). CASA uses slides with unique chambers (e.g., GoldCyto®, Leja, etc., disposable rooms). However, since these special slides are too expensive for some laboratories, glass slides with cover slip are used instead of special slides. However, sperm counts can be underestimated or overestimated, depending on the types of products used. Calibration of devices for measuring concentration is critical to ensure correct sperm count per dose and to produce maximum quantities per ejaculation. Standardizing a laboratory procedure for assessing sperm concentration is influenced by factors such as animal species, sample size needed, frequency of operations, number of samples evaluated per day, and cost of operation. (11). This study aims to determine the accuracy of concentration measurements made with glass slides compared to GoldCyto® chambered slides on CASA.

## Materials and methods

### Semen samples

GoldCyto® slides and glass slides, which can be used in the Computer-Assisted Sperm Analyzer (CASA), (SCA®, Microptic, Barcelona, Spain) were compared with the spectrophotometer concerning the accuracy of the evaluation. It was studied with a sample with a known reference range as the control group. The study’s control group consisted of known-concentration samples (72–80 million sperm/mL) measured with a spectrophotometer purchased from the Republic Of Turkey Ministry Of Agriculture And Forestry International Center For Livestock Research and Training. For this purpose, 21 cryopreserved straws from the same date from the same bull were thawed at 37°C. Each thawed straw was divided equally into two parts and used in the study groups: GoldCyto® slides and glass slides.

### Determination of sperm concentration

Straws were used in the control group, which the precision was measured (72–80 million sperm/mL) with a spectrophotometer to compare the measurement accuracy of the study groups.

### Spectrophotometer analysis

A spectrophotometer is the most widely used method for sperm concentration assessment in animal semen processing centers (9). In this study, the sperm concentration of fresh semen collected from a bull was evaluated by a spectrophotometer (Accucell bovine photometer (IMV), France). After being diluted to a final semen concentration of 72–80 million sperm/mL, the semen was packaged in 0.25 mL straws and frozen. Twenty-one frozen semen straws were used for semen density determination in GoldCyto® slide and glass slide groups.

### GoldCyto® slides

GoldCyto® slides (20-micron depth) were used for the analysis to be performed with the CASA software [Sperm Class Analyzer (SCA® v.6.5.0.91, Barcelona, Spain)]. Before evaluation, the value of 5 μL was entered into the software according to the procedure (Sperm Class Analyzer, Microptic, Barcelona, Spain), and the same amount of thawed semen sample was placed on the GoldCyto® slides. Then measurements were taken in 5 different areas and evaluated in the CASA software.

### Glass slides

Concentration measurements with a 76 × 26 mm glass slide and glass slip were done the same way by analysis in the CASA software. The analysis was performed by placing 5 μL of sperm on the slide and mounting it with a 22 × 22 mm coverslip. The information that 5 μL was given to the program in the CASA software was entered according to the procedure. Then measurements were taken in 5 different areas and evaluated in the CASA software.

### Statistical analysis

The SPSS 13.0 Package program was used to analyze the data obtained in our study. One sample Test was used to compare the data of sperm concentration groups with each other. The statistical significance level of the differences between the groups was given as *p* < 0.05.

## Results

**Figure 1:**
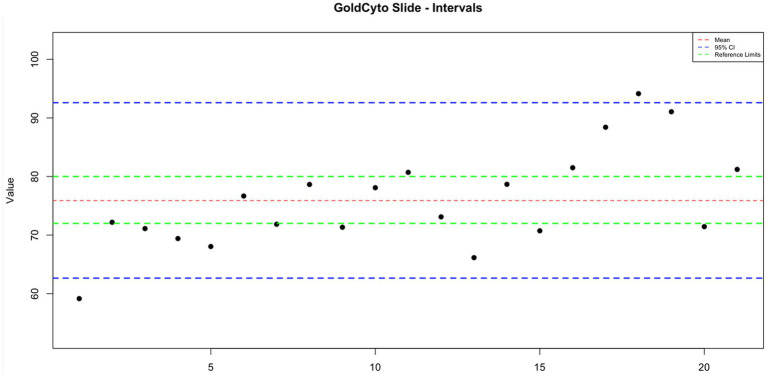
The interval plot for the measurements of the GoldCyto® slides.

**Figure 2:**
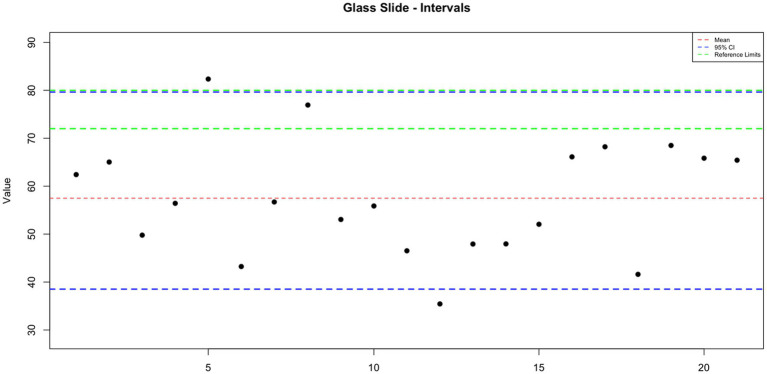
The interval plot for the measurements of the glass slides.

GoldCyto® slides and glass slides compared with a sample with a known reference range (72–80 million sperm/mL) as the control group were 75.887 ± 1.844 and 57.498 ± 2.617, respectively. In Table 1, the concentration values of both counting systems are shown. There were no significant differences in concentration results between GoldCyto® slides (*p* > 0.05) and the spectrophotometer. However, the counting chamber of the used glass slide had a significantly lower measured concentration (*p* < 0.001). The interval plots for the measurements of the GoldCyto® slides and glass slides are shown in Figures 1, 2.

**Table 1:** Comprasion of sperm concentration assesed using GoldCyto® slides and glass slides.

Counting chamber	Concentration (x10^6^sperm/mL)	Std. Error mean	*p*
Control group (Spectrophotometric Analysis)	72–80		
GoldCyto® Slides	75.887	1.844	>0.05
Glass Slides	57.498	2.617	<0.001

## Discussion

The sperm concentration should be accurately determined not only in clinical infertility examination but also while attempting to determine total sperm production in the testis (12). Even though all commonly preferred concentration determination methods are proven procedures, each technique has produced different results. Current research has found interlaboratory coefficients of variation (CV) for sperm concentration values ranging from 23 to 73%, 53 to 80%, and 21 to 34%. This situation highlights the challenge of comparing results from different laboratories and estimating scientific research (13, 14).

According to Brito et al. (9) spectrophotometer analysis of sperm is rapid, takes a minimal amount of material, and is economical in terms of equipment and consumables. Furthermore, they indicated that it is the most widely used method for determining fresh sperm concentration because the CV, which reports precision in scientific research, is between 3 and 6% (9). In this direction, the control group of our study consisted of semen straws whose spectrophotometer concentrations were determined.

Bompart et al. (15) revealed that CASA technology needs the usage of certain counting rooms, and the properties of each room type need to be known. Currently, disposable capillary-loaded counting chambers with a depth of 20 μm are used in CASA systems to analyze spermatological characteristics. For animal semen analysis, GoldCyto® slides are disposable slides created exclusively for concentration evaluation with CASA systems.

In current CASA systems, the 20 μm deep chambers provide a single cell layer. The particles suspended in the fluid are exposed to a velocity gradient when the chamber is filled by capillary action, causing them to migrate perpendicular to the flow direction. The viscosity, which dominates the medium in a liquid flow while reaching its peak velocity in the middle of the partition walls, reduces to zero at the partition walls. The velocity gradient causes the suspended particles to have transverse buoyancy, the force pushing them toward two fixed planes of a short, calculable distance from the walls. This situation has been revealed previously and is called the Segre-Silberberg (SS) effect (16). The SS effect causes cell concentration fluctuations in the chamber flow by attracting particles from the flow margins to the faster-flowing regions of the liquid (9). Based on this, it is thought that this may be the reason for the different results obtained in this study in both glass slides and GoldCyto® slides were due to the SS effect. The accuracy of CASA has been extensively studied; however, the results were inconsistent with each other.

The accuracy of concentration estimates derived *via* CASA is affected by chamber design and sperm preparation factors, in addition to hardware and software. The variations in CASA results can be explained by the failure to effectively eliminate non-spermatozoan particles, which seemed too minute and too brilliant to be detected by the system, sperm clumping, and improper sample dilution to attain the optimum concentration (9). This situation can be explained because different values were obtained from concentration measurements done with GoldCyto® slides and glass slides in this study.

In an investigation of andrology facilities in Italy, Filimberti et al. (17) discovered considerable variability in semen analysis results. According to the findings of this study, numerous optimizations were done in laboratories utilizing the CASA system. This condition, however, results in data disagreement between laboratories. The findings of this study confirm our study. Valid optimization studies are needed for optimal results. Despite the objectivity of CASA system evaluations, Dardmeh et al. (18) suggest that when different commercially available slides are used, there are still some variations in the results of sperm concentration, motility, and other kinematic parameters. These results are in agreement with our present study.

Using a computer-assisted semen analyzer and several imaging chambers, Lenz et al. (19) compared the quality of bovine seminal samples. The study found that if the laboratory worker utilized various slide chambers when analyzing semen, a wide range of values or a narrower range of motile sperm percentages may be produced.

For reliable findings with disposable chambers, strict technical requirements must be observed, whether performing manual counts or utilizing CASA. These include verified chambers, cell immobilization, the SS correction factor, and the use of DNA fluorescent probes (for CASA) (9); the same situation also applies to the glass slide. As a result, the evaluator must ensure that the results are correct at each semen examination, and their analyses must be repeatable. It should also make sure that it is comparable to other methods and or other slide chambers.

Today counting chambers loaded with disposable capillaries with a depth of 20 μm are mostly used in CASA systems. There is no strict gold standard for semen analysis with the CASA system in animals and humans, and different CASA systems and counting chambers are used worldwide. According to the data we obtained, the concentration measurements done using glass slides were statistically significantly lower than those of the spectrophotometer and GoldCyto® slides (*p* < 0.001). The fact that GoldCyto® slides are disposable has a high cost, and the motility decreases due to capillary action argues for the use of glass slide in laboratory conditions. However, according to the studies performed, the concentration measurement results using glass slides are not consistent with other methods, and the data from our study support this. The lower concentration using glass slides may be due to the Segre-Silberberg effect. It was concluded that GoldCyto® slides are more suitable instead of glass slides in the concentration examinations of semen. Therefore, further studies are needed to optimize the use of glass slides.

## Data availability statement

The original contributions presented in the study are included in the article/supplementary material, further inquiries can be directed to the corresponding author.

## Ethics statement

Ethical approval was not required for the studies on animals in accordance with the local legislation and institutional requirements because only commercially available established cell lines were used.

## Author contributions

EA: Writing – original draft.
